# Lenticular cytoprotection. Part 1: The role of hypoxia inducible factors-1α and -2α and vascular endothelial growth factor in lens epithelial cell survival in hypoxia

**Published:** 2013-01-02

**Authors:** Sudha Neelam, Morgan M. Brooks, Patrick R. Cammarata

**Affiliations:** Department of Cell Biology and Anatomy, University of North Texas Health Science Center at Fort Worth, Fort Worth, TX

## Abstract

**Purpose:**

The prosurvival signaling cascades that mediate the unique ability of human lens epithelial cells to survive in their naturally hypoxic environment are not well defined. Hypoxia induces the synthesis of the hypoxia inducible factor HIF-1α that in turn, plays a crucial role in modulating a downstream survival scheme, where vascular endothelial growth factor (VEGF) also plays a major role. To date, no published reports in the lens literature attest to the expression and functionality of HIF-2α and the role it might play in regulating VEGF expression. The aim of this study was to identify the functional expression of the hypoxia inducible factors HIF-1α and HIF-2α and establish their role in regulating VEGF expression. Furthermore, we demonstrate a link between sustained VEGF expression and the ability of the hypoxic human lens epithelial cell to thrive in low oxygen conditions and resist mitochondrial membrane permeability transition (also referred to as lenticular cytoprotection).

**Methods:**

Hypoxia inducible factor translation inhibitors were used to demonstrate the role of HIF-1α and HIF-2α and the simultaneous expression of both hypoxic inducible factors to determine their role in regulating VEGF expression. Axitinib, which inhibits lenticular cell autophosphorylation of its VEGF receptor, was employed to demonstrate a role for the VEGF–VEGFR2 receptor complex in regulating Bcl-2 expression. Specific antisera and western blot analysis were used to detect the protein levels of HIF-1α and HIF-2α, as well as the proapoptotic protein, BAX and the prosurvival protein, Bcl-2. VEGF levels were analyzed with enzyme-linked immunosorbent assay (ELISA). The potentiometric dye, 5,5′,6,6′-tetrachloro1,1′,3,3′-tetraethyl-benzimidazolylcarbocyanine iodide, was used to determine the effect of the inhibitors on mitochondrial membrane permeability transition.

**Results:**

Cultured human lens epithelial cells (HLE-B3) maintained under hypoxic condition (1% oxygen) displayed consistent accumulation of VEGF throughout the 72 h incubation period. Using hypoxia inducible factor translation inhibitors targeting HIF-1α or HIF-2α, the specific inhibition of each protein did not diminish VEGF synthesis. The combined inhibition of HIF-1α and HIF-2α expression, using a double hypoxia inducible factor translation inhibitor, markedly decreased the level of VEGF. The inhibition of VEGF synthesis was associated with a profound deficiency in the level of the prosurvival protein, Bcl-2. Axitinib also prevented the VEGF-mediated expression of Bcl-2. The loss of VEGF coupled with the decrease in intracellular Bcl-2 correlated with marked mitochondrial depolarization, an early predictor of cellular apoptosis.

**Conclusions:**

Our data support a model in which the sustained synthesis of VEGF in human lens epithelial cells, maintained under hypoxic condition, is regulated by a compensatory inter-relationship between HIF-1α and HIF-2α. VEGF acts as a prosurvival factor in hypoxic lens epithelial cells by maintaining consistent expression of the prosurvival protein Bcl-2, which likely prevents the translocation of cytosolic BAX to the outer mitochondrial membrane, thus preventing the initiation of mitochondrial depolarization.

## Introduction

The lens exists in a natural state of hypoxia [1]. The state of severe oxygen deprivation, an environment to which the lens is uniquely adapted, would be detrimental to most other cell types. Indeed, the lens has developed several unique survival mechanisms enabling it to thrive in a chronically hypoxic environment and to oppose oxidative injury [2-4]. Despite such knowledge, however, relatively little is known regarding how human lens epithelial cells (HLECs) regulate their inherent signal transduction mechanisms to thrive in a hypoxic environment of less than 5% oxygen and prevent mitochondrial membrane permeability transition (mMPT), a cellular event that under normal circumstances precludes the onset of apoptosis and cell death.

The status quo regarding the role that vascular endothelial growth factor (VEGF) plays in lens cell proliferation is that VEGF is one of several factors that stimulate lens cell proliferation and promote fiber differentiation [5]. Although such a multifaceted role for VEGF is generally accepted, a mechanism-based understanding of the signal transduction pathways that are involved in regulating lenticular cellular homeostasis in hypoxia is unknown. To date, published studies largely support a role for hypoxia inducible factor-1α (HIF-1α) as the transcription factor that controls VEGF expression in hypoxia, but there are inconsistencies in the lens literature. HIF-1 is recognized as an age-dependent regulator of lens cell proliferation in the hypoxic lens and is known to degrade under conditions in or above atmospheric oxygen [6]. Additionally, Garcia et al. [7] have demonstrated that VEGF continues to be synthesized in the hypoxic lens in the absence of HIF-1. In other words, there is a continuous expression of VEGF, in lens or cultured lens epithelial cells, whether it is in hypoxia or atmospheric oxygen, in the absence of HIF-1α, suggesting that additional regulatory proteins are at play.

The hypoxia inducible transcription factors HIF-1α and HIF-2α are heterodimeric α subunits that dimerize with constitutively expressed β subunits [8,9]. They bind to the hypoxia responsive elements and induce the transcription of genes regulating proliferation, angiogenesis, and growth factors, such as VEGF and erythropoietin (EPO) [10,11]. Although HIF-1α and HIF-2α are similar in structure and are activated by hypoxia, they are not redundant in function [12]. For example, a human embryonic kidney cell line (HEK293) that has been shown to express stabilized forms of HIF-1α and HIF-2α [13], and that the HIF-1α and HIF-2α transcription factors have unique gene targets. As this observation relates to the study presented herein, HIF-1α and HIF-2α have also been shown to regulate VEGF expression under hypoxia [14,15], but this does not exclude the likely possibility that the two transcription factors have divergent gene targets. Using MCF-7 cells (a breast carcinoma cell line) in conjunction with small interfering RNA knockdown, Carroll et al. [16] previously showed that HIF-1α and HIF-2α regulate VEGF expression under hypoxia. Diminishing the expressed levels of HIF-2α resulted in a reciprocal increase in the expression of HIF-1α, maintaining VEGF expression. The data complement the findings presented in this study in that we also report a reciprocal relationship between the two hypoxia inducible factors in regulating VEGF expression. A role for HIF-2α in regulating VEGF expression has not previously been reported in the lens epithelium. Our data add an important new dimension to lenticular science in that we agree with Carroll et al.’s conclusions [16]: we also would “predict that targeting both HIF-1α and HIF-2α in cells that show this reciprocal response in hypoxia would be desirable for blocking VEGF expression.”

Using a pharmacological approach with specific hypoxia inducible factor translation inhibitors, we demonstrate that diminishing the levels of HIF-1α and HIF-2α in concert but not HIF-1α or HIF-2α alone is associated with diminished levels of VEGF and a corresponding decrease in the level of the prosurvival protein, Bcl-2, associated with the onset of mitochondrial depolarization. The studies presented will establish the novel finding that the survival of human lens epithelial cells (HLECs) in hypoxia depends on the uninterrupted intracellular manifestation of VEGF controlled by the compensatory expression of the hypoxia inducible factors HIF-1α and HIF-2α. Further, we reconfirm our observations by applying axitinib, a selective inhibitor of VEGF receptor kinases 1–3 [17]. The consequence of the use of axitinib is to effectively remove the action of VEGF from the cell culture system, which resulted in the simultaneous elimination of Bcl-2, consistent with a role for VEGF–VEGF receptor (VEGFR2) in the Bcl-2 regulation pathway. Collectively, these results provide compelling evidence of an important relationship between levels of HIF-1α and HIF-2α and VEGF in lens epithelial cells and the relationship with a prosurvival activity of Bcl-2 and mitochondrial function.

## Methods

### Materials

Topotecan hydrochloride, a recognized topoisomerase I inhibitor, was purchased from AvaChem Scientific LLC (San Antonio, TX). HIF-1α translation inhibitor (KC7F2), HIF-2α translation inhibitor (CAS882268–69–1), and the HIF-1α and HIF-2α double translation inhibitor (FM19G11) were purchased from EMD Chemicals (Billerica, MA). Axitinib (N-methyl-[[3[(1E)-2-(2-pyridinyl)ethenyl]-1H-indazol-6-yl]thio]-benzamide) was purchased from Tocris Bioscience (Ellisville, MO). Mitogen activated protein kinase 1/2 inhibitor (MEK1/2), UO126 (1,4-diamino-2, 3-dicyano-1, 4-bis [2-aminophenylthio] butadiene), and phosphoinositide (PI) 3-kinase inhibitor LY294002 were purchased from Cell Signaling Technology (Danvers, MA). All inhibitors were dissolved in dimethyl sulfoxide (DMSO). The final concentration of DMSO for all the inhibitor assays did not exceed 0.05%.

### Cell cultures

HLE-B3 cells, a human lens epithelial cell line immortalized by the SV-40 virus, were obtained from U. Andley (Washington University School of Medicine, Department of Ophthalmology, St. Louis, MO). Cells were maintained in minimal essential media (MEM) containing 5.5 mM glucose supplemented with 20% fetal bovine serum (FBS; Hyclone Laboratories, Logan, UT), 2 mM L-glutamine, nonessential amino acids, and 0.02 g/l gentamycin solution (Sigma Chemical, St. Louis, MO) and maintained at 37 °C and 5% CO_2_. All experiments were performed with monolayers of HLE-B3 cells that did not exceed passage 23. Cells were generally maintained in 20% FBS (Gemini Bio-Products, Sacramento, CA) MEM for 24–48 h and then switched to 10% MEM for 24 h with a final medium change to serum-free MEM 24 h before the day of the experiments. For all experiments involving inhibitor treatment, a parallel control set of cells mock-treated with a concentration of DMSO no greater than 0.05% was included.

### Western blot analysis

Total cell lysates were collected from HLE-B3 cultures after treatment by rinsing adherent cells with ice-cold 1× phosphate buffered solution (stock buffer: 150 mM sodium chloride, 59 mM sodium phosphate monobasic, 49 mM sodium phosphate dibasic, pH 7.4) and then adding hot lysis buffer (0.12 M Tris HCl (pH 6.8), 4% sodium dodecyl sulfate (SDS), and 20% glycerol, 280 μl boiled to 100 °C) directly to cell monolayers. Lysates were collected and sonicated for 5 s, and a portion of the sample was removed to determine the protein concentration. Protein concentration was determined using the EZQ protein quantification kit from Invitrogen (Carlsbad, CA); 3× SDS (Laemmli) buffer was added to the lysates, which were subsequently boiled for 5 min; and the proteins were resolved by electrophoresis on 12% SDS-polyacrylamide gels (20 μg protein/lane). Proteins were then transferred to nitrocellulose (Scheicher and Schuell, Keene, NH).

For western blot analysis, nitrocellulose membranes were blocked with Tris-buffered saline (TBS, 1% BSA, and 0.02% Tween−20 in Tris–buffered saline) for 60 min. These membranes were probed overnight at 4 °C with primary antibodies. The blots were then rinsed in TBS (4× with 5-min washes) and incubated in either goat anti-rabbit horseradish peroxidase conjugate or goat anti-mouse horseradish peroxidase conjugate (Santa Cruz Biotechnology, Santa Cruz, CA) for 1 h at room temperature. Required concentrations of antibodies were determined according to the manufacturer's suggested protocols. Blots were again rinsed in TBS (4 × 5 min washes), and proteins were detected using a SuperSignal west pico chemiluminescent kit from Pierce (Rockford, IL).

Primary antibodies used in this study were rabbit anti-BAX (Cell Signaling Technology), rabbit anti-Bcl-2 (Cell Signaling Technology), rabbit anti-actin (Santa Cruz Biotechnology), rabbit anti-HIF-1α (Bethyl Laboratories Inc., Montgomery, TX), rabbit anti-HIF-2α (Novus Biologicals, Littleton, CO), mouse anti-phospho-p44/42 mitogen-activated protein kinase (Thr202/Tyr204; Cell Signaling Technology, Danvers, MA), and rabbit anti-phospho-Akt (Ser473; Cell Signaling Technology). Western blot analysis was repeated at least twice, and in many cases, three times, in all experiments.

### Enzyme-linked immunosorbent assay for vascular endothelial growth factor

An enzyme-linked immunosorbent assay (ELISA) was used to detect secreted levels of VEGF. VEGF was determined using the Invitrogen VEGF ELISA kit (Grand Island, NY). HLE-B3 cells were cultured in 25 cm^2^ tissue culture flasks in 20% FBS, and transferred 24 h in serum-free media before the experiment was initiated. Flasks were set up in triplicate for 3, 8, or 24 h of cell incubation in hypoxia (1% oxygen) or atmospheric oxygen (21% oxygen). Cell-free supernatants were collected and analyzed according to the manufacturer’s instructions, and optical density at 450 nm was determined using a Molecular Devices SpectraMax 190 (Sunnyvale, CA).

### Analysis of mitochondrial membrane damage

After experimental treatments, cells were stained with the cationic dye 5,5′,6,6′-tetrachloro1,1′,3,3′-tetraethyl-benzimidazolylcarbocyanine iodide (JC-1; Molecular Probes, Eugene, OR) as described previously [18] to visualize the state of mitochondrial membrane potential. JC-1 is a potentiometric dye that exhibits a membrane potential dependent loss as J-aggregates (polarized mitochondria) to accumulation of JC-1 monomers (depolarized mitochondria) as indicated by the fluorescence emission shift from red to green [18]. That is, mitochondrial depolarization is indicated by an increase in the green-to-red fluorescence intensity ratio.

The cells were stained using the following procedure. Monolayers were rinsed one time with serum-free MEM. Cell monolayers were incubated with serum-free MEM containing 5 μg/ml JC-1 at 37 °C for 30 min. After this incubation, cells were again rinsed two times with the serum-free MEM, and multiple images were obtained using a 10× objective on a confocal microscope (Zeiss LSM 410, Thornwood, NY) excited at 488 nm set to simultaneously detect green emissions (510–525 nm) and red emissions (590 nm) channels using a dual band-pass filter.

### Statistics

The images from the JC-1 analysis were taken and separated into individual red and green channels using ImageJ software (NIH, Rockville, MD). The background fluorescence was removed from each image. The fluorescence intensity signal from each image was quantified for the entire image and expressed as the ratio of the green intensity over the red intensity. For ELISA, a Student *t* test (the topotecan experiments) and a one-way ANOVA (ANOVA; the HIF translation inhibitor experiments) were performed using the software from GraphPad Prism, version 5.00 (La Jolla, CA).

## Results

### Vascular endothelial growth factor expression in HLE-B3 cells in hypoxia and atmospheric oxygen

**Figure 1:**
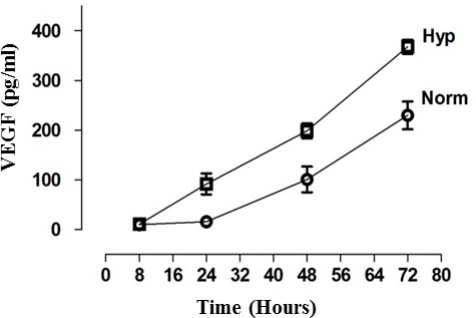
Sustained and cumulative expression of VEGF in hypoxia and atmospheric oxygen. Detection of VEGF levels in hypoxia with ELISA. HLE-B3 cells were cultured in 25 cm^2^ flasks with 20% FBS and switched to serum-free media 24 h before the experiment. The cells were incubated with 3 ml of serum-free media in hypoxia (1% oxygen) or remained in atmospheric oxygen (about 21% oxygen) for up to 72 h. Cell-free supernatants were collected in triplicate at 8, 24, 48, and 72 h and analyzed with ELISA to detect the VEGF levels. VEGF consistently accumulated throughout the 72 h incubation period regardless of whether the cells were maintained in hypoxia or atmospheric oxygen (p<0.05). A Student *t* test was performed to compare the VEGF levels between hypoxia and normoxia. Significantly higher levels of VEGF were detected at all-time points beyond the initial 8h point in hypoxia compared with atmospheric oxygen. Error bars are not shown at time points because the symbol is larger than the error bar.

The levels of VEGF synthesized by HLE-B3 cells maintained in either hypoxia or atmospheric oxygen were compared. HLE-B3 cells were serum-starved for 24 h, then fresh serum-free media were introduced, and cell cultures switched to hypoxia (1% oxygen) or allowed to remain in atmospheric oxygen (about 21% oxygen) for up to 72 h. Cell-free supernatants were collected in triplicate at 8, 24, 48, and 72 h. The VEGF levels in the supernatants were analyzed with ELISA. VEGF steadily accumulated regardless of whether the cells were maintained in hypoxia or atmospheric oxygen throughout the duration of the time course. Significantly higher amounts of VEGF, at all collected time points beyond the initial 8 h point, were evident in hypoxia-maintained cells relative to the corresponding collected time point from atmospheric oxygen (Figure 1).

### Inhibition of HIF-1α by topotecan

**Figure 2:**
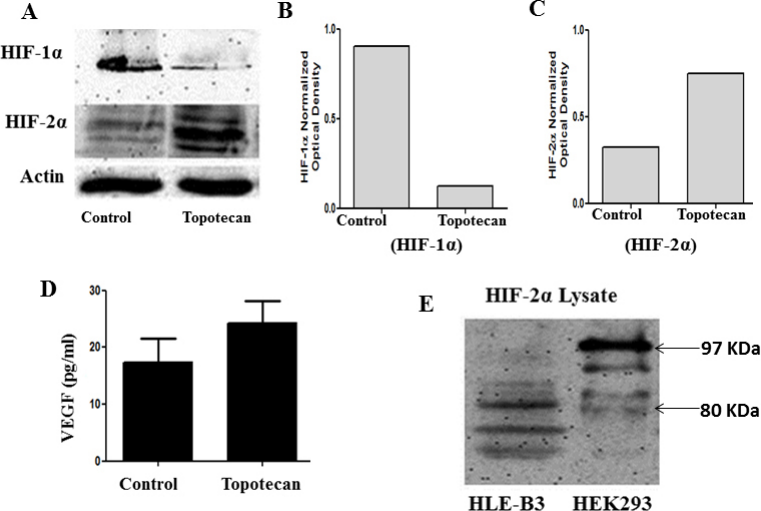
HIF-1α inhibition does not affect VEGF expression. **A**: western blot analysis of HIF-1α expression in HLE-B3 cells treated with topotecan. Cell lysates were collected from cells treated with 500 nM topotecan in 0.01% DMSO after 8 h of hypoxic incubation. Control cells were mock treated with 0.01% DMSO and maintained in hypoxia as the topotecan-treated cells. Twenty μg protein/lane of cell lysates were analyzed with western blot analysis, and lane loading was normalized using a 1:1,000 dilution of rabbit anti-pan-actin antibody. Topotecan inhibited the expression of HIF-1α (1:1,000 dilution of rabbit anti- HIF-1α antibody) while a compensatory increase in HIF-2α was noted (1:1,000 dilution of rabbit anti- HIF-2α antibody). **B, C**: Densitometry analysis of HIF-1α and HIF-2α, respectively. **D**: Effect of HIF-1α inhibition on VEGF synthesis in hypoxia. HLE-B3 cells were cultured in 25 cm^2^ flasks with 20% FBS and switched to serum-free media 24 h before the experiment. The cells were incubated with 3 ml of serum-free media containing 500 nM topotecan or 0.01% DMSO for 8 h of hypoxic exposure. Cell-free supernatants were collected in triplicate at the end of hypoxic incubation and analyzed for VEGF levels with ELISA. There was no significant difference in the VEGF levels between the topotecan-treated cells and control cells. (A Student *t* test was performed to compare the VEGF levels between control and treated sample, and the p value was greater than 0.05.) **E**: HIF-2α protein from HLE-B3 control cells compared with a standard lysate prepared from human embryonic kidney cells (HEK) 293 (Novus Biologicals, Litton, CO). The HIF-2α found in the standard lysate migrated at about 97 kDa. The HIF-2 α from HLE-B3 cells was about 80 kDa.

Topotecan inhibits topoisomerase-I, thus preventing HIF-1α transcription and protein accumulation in hypoxia [19,20]. HLE-B3 cells were serum-starved for 24 h after which 500 nM topotecan (dissolved in 0.01% DMSO) was added to the cells. Control cells were mock-treated by incubation in serum-free media with 0.01% DMSO. Hypoxic cell lysates were collected after 8 h of topotecan treatment for western blot analysis of HIF-1α and HIF-2α expression. At the same time, cell-free supernatants were collected in triplicate and analyzed with ELISA to determine the VEGF levels. HIF-1α is a transcription factor, expressed and stabilized in hypoxia, that thus plays a major role in regulating VEGF synthesis. Western blot analysis of topotecan-treated lysates displayed a marked decrease in the protein levels of HIF-1α relative to the control cells (Figure 2A). The attenuation of HIF-1α did not affect VEGF synthesis relative to the untreated cells (Figure 2D). HIF-2α has been shown to induce VEGF synthesis in other cell systems [21,22], but a role for HIF-2α in regulating VEGF synthesis in human lens epithelial cells has never been reported. Topotecan-treated samples showed a marked increase in HIF-2α expression relative to the control cells (Figure 2A). Thus, elevated expression of HIF-2α was initiated as a result of the loss of HIF-1α by topotecan treatment.

### HIF-2α standard lysates

Western blot analysis of HIF-2α protein expression revealed a set of three bands of approximate molecular weight of 80 kDa. Foorogian et al. [23] reported HIF-2α as a set of three bands of approximate molecular weight of 100 kDa in retinal pigment epithelial cells. To further confirm the identity of HIF-2α in HLE-B3 cells, a HIF-2α standard lysate (Novus Biologicals) was compared with lysate from HLE-B3 cells. Whereas the HIF-2α bands in HLE-B3 cells were of approximate molecular weight, 80 kDa, the standard lysate was of approximate molecular weight 97 kDa (Figure 2E).

### Cytoprotection in HLE-B3 cells is not affected by the loss of HIF-1α

**Figure 3:**
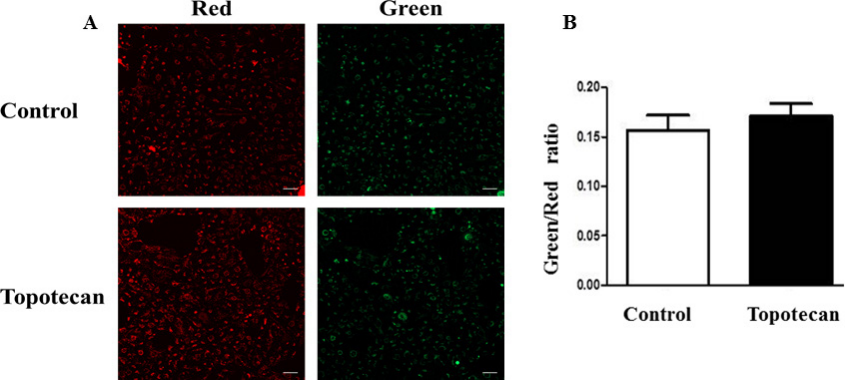
Loss of HIF-1α does not influence mitochondrial membrane potential. JC-1 analysis of HLE-B3 cells treated with topotecan. Cells were incubated in serum-free media containing 500 nM of topotecan in 0.01% DMSO for 3 h in hypoxia. Control cells were treated with 0.01% DMSO in serum-free media and likewise exposed for 3 h in hypoxia. After 3 h of hypoxic exposure, fresh, oxygenated media without the inhibitor but with the addition of 5 µg/ml of JC-1 dye were added and incubated at 37 °C for 30 min. JC-1 is a potentiometric dye that exhibits a membrane potential dependent loss as J-aggregates (polarized mitochondria) when transitioned to JC-1 monomers (depolarized mitochondria), as indicated by a fluorescence emission shift from red to green. Therefore, mitochondrial depolarization can be indicated by an increase in the green/red fluorescence intensity ratio. The media were removed, and fresh serum-free media without inhibitor or potentiometric dye were again added to the cells. **A**: Confocal imaging of mitochondrial membrane depolarization after inhibition of HIF-1α. Note the proportionally equivalent red and green fluorescence between topotecan-treated and mock-treated cells, indicating that the membrane potential was not altered by inhibiting HIF-1α expression. These images were taken from a randomly chosen field. (The bar represents 20 µm.) **B**: There was no significant difference in the green/red fluorescence ratio between the control and topotecan-treated cells. Student *t* test, p>0.05.

The effect of inhibition of HIF-1α on mitochondrial membrane permeability transition (i.e., lenticular cytoprotection) was evaluated with the JC-1 assay. Cells were incubated with 500 nM topotecan for 3 h in hypoxia and switched to atmospheric oxygen. The hypoxic media were replaced with fresh, oxygenated media without an inhibitor, and 5 µg/ml of JC-1 dye was introduced to the cell cultures and incubated at 37 °C for 30 min. The media were removed, and fresh media without inhibitor or potentiometric dye were added to the cells. Control cells were incubated in serum-free media with DMSO but were otherwise treated identically. There was no significant difference in the green-to-red fluorescence ratio between the control cells and the cells treated with topotecan (Figure 3).

### Inhibition of hypoxia inducible factors by translation inhibitors

**Figure 4:**
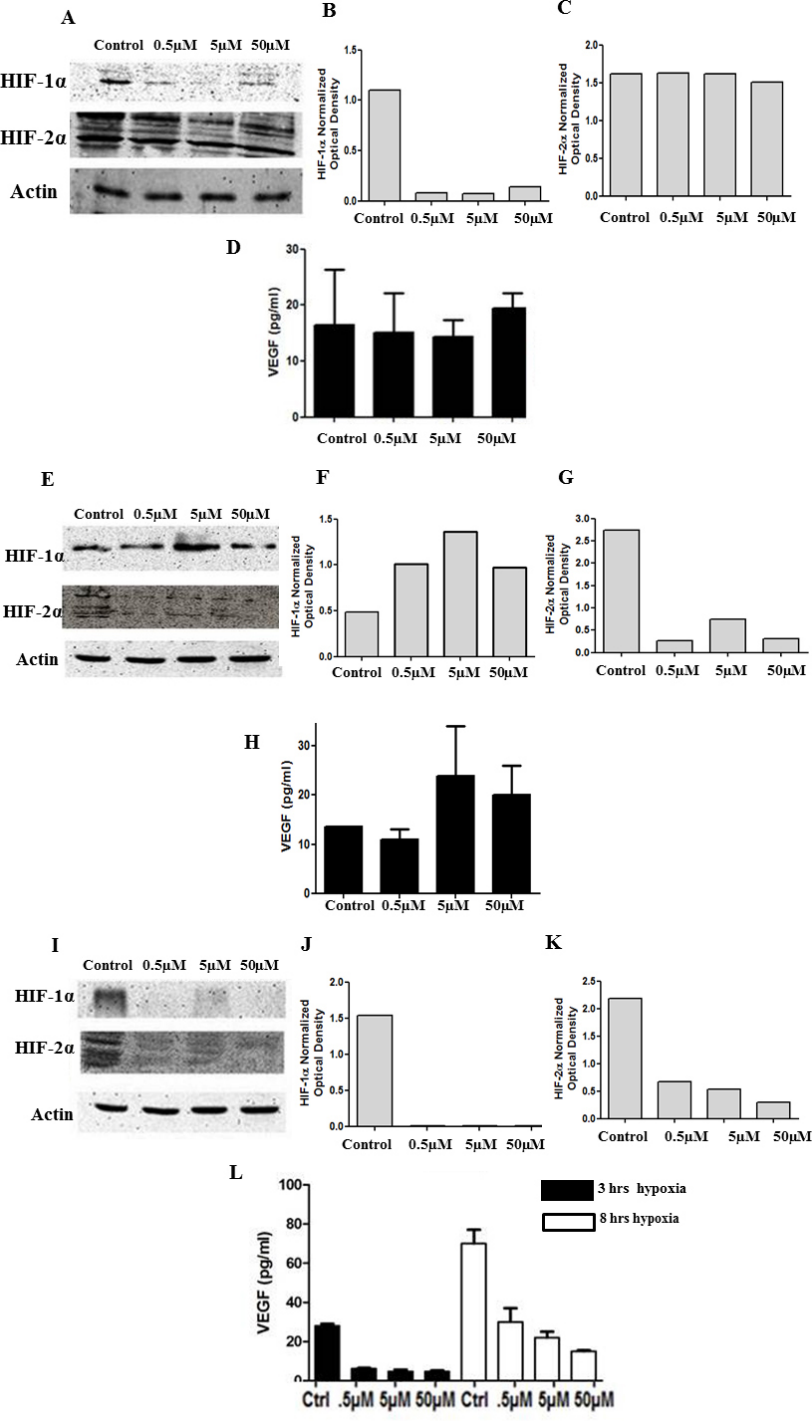
Inhibition of both HIF-1α and HIF-2α elicits the loss of VEGF expression. HLE-B3 cells were cultured in 25 cm^2^ flasks with 20% FBS and switched to serum-free media 24 h before the experiment. The cells were incubated with 3 ml of serum-free media containing 0.5 µm, 5 µm, and 50 µm HIF-1α inhibitor, HIF-2α inhibitor, and HIF-1α/HIF-2α double translation inhibitor for 3 or 8 h in hypoxia. The effect of the inhibitors on HIF-1α and HIF-2α protein expression was analyzed using western blot analysis. Cell lysates (20 ug protein/lane) were identified using either anti-rabbit HIF-1α or HIF-2α at 1:1000 dilutions and lane loading was normalized using a 1:1000 dilution of rabbit anti- pan-actin antibody. Effect of HIF-1α and/or HIF-2α inhibition on VEGF levels in hypoxia. HLE-B3 cells were cultured in 25 cm^2^ flasks with 20% FBS and switched to serum-free media 24 h before the experiment. The cells were incubated with 3 ml of serum-free media containing 0.5 µm, 5 µm and 50 µm HIF-1α inhibitor, HIF-2α inhibitor and HIF-1α/HIF-2α double translation inhibitor for 3 or 8 h in hypoxia. Cell free supernatants collected in triplicate were analyzed for VEGF levels by ELISA. The HIF-1α translation inhibitor at all concentrations inhibited HIF-1α without affecting the HIF-2α protein synthesis (**A**). Figure (**B**) and (**C**) represent the densitometry analysis for HIF-1α and HIF-2α protein expression. There was no significant difference in the VEGF levels between the control cells and cells treated with HIF-1α inhibitor (**D**). One-way ANOVA analysis was performed to compare the VEGF levels between the control and the three concentrations of HIF-1α inhibitor and the p value was >0.05. The HIF-2α translation inhibitor at all concentrations inhibited HIF-2α without affecting the HIF-1α protein synthesis (**E**). Figure (**F**) and (**G**) represent the densitometry analysis for HIF-1α and HIF-2α protein expression. There was no significant difference in the VEGF levels between the control cells and cells treated with HIF-2α inhibitor (**H**). One -way ANOVA analysis was performed to compare the VEGF levels between the control and the three concentrations of HIF-2α inhibitor and the p value was >0.05.The HIF-1α/HIF-2α double translation inhibitor at all concentrations inhibited HIF-2α and HIF-1α protein synthesis (**I**). Figure (**J**) and (**K**) represent the densitometry analysis for HIF-1α and HIF-2α protein expression. There was significant difference in the VEGF levels between the control cells and cells treated with HIF-1α/ HIF-2α double translation inhibitor at 3 h and 8 h of hypoxia. **L**: One-way ANOVA analysis was performed to compare the VEGF levels between the control and the three concentrations of HIF-1α/ HIF-2α double translation inhibitor and the p value was <0.05.

The role of hypoxia inducible factors in regulating VEGF expression was further evaluated with specific translation inhibitors. KC7F2 is a specific HIF-1α translation inhibitor that suppresses HIF-1α protein synthesis but not mRNA transcription [24]. CAS882268–69–1 is a specific HIF-2α translation inhibitor that suppresses HIF-2α protein synthesis but not mRNA transcription [25,26]. FM19G11 is a histone acetylase inhibitor shown to simultaneously inhibit HIF-1α and HIF-2α transcription activation [27]. Cells were maintained in serum-free media for 24 h and treated in the presence and absence of one of three conditions: 0.5 µm, 5 µm, and 50 µm of the HIF-1α translation inhibitor, KC7F2, the HIF-2α translation inhibitor, CAS882268–69–1, or the HIF-1α/HIF-2α double translation inhibitor, FM19G11, dissolved in 0.05% DMSO for 3 h in hypoxia. Control cells were incubated in serum-free media with 0.05% DMSO. Cell-free supernatants were collected in triplicate at the completion of the 3 h exposure period. The supernatants were analyzed for VEGF levels with ELISA. The inhibition of HIF-1α and HIF-2α by the translation inhibitors was confirmed with western blot analysis (Figure 4). Inhibition of HIF-1α or HIF-2α by their respective specific translation inhibitors did not influence VEGF synthesis in hypoxia relative to the relevant controls (Figure 4D,H). In contrast, the simultaneous inhibition of HIF-1α and HIF-2α caused a significant decrease in VEGF synthesis (Figure 4L). To further confirm the sustained decrease in VEGF accumulation in the presence of the double translation inhibitor, cells were treated for up to 8 h in hypoxia. The prolonged exposure to FM19G11 continued to show a significant decrease in VEGF levels relative to the control cells (Figure 4L).

### Cytoprotection in HLE-B3 is affected by the combined loss of HIF-1α and HIF-2α

**Figure 5:**
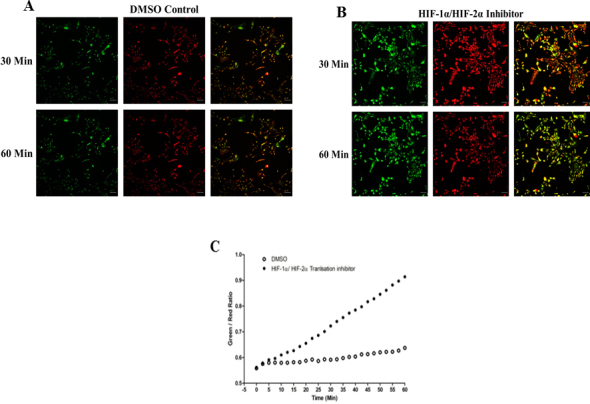
Inhibition of both HIF-1α and HIF-2α elicits mitochondrial membrane depolarization. Cells were treated with 50 μM of HIF-1α/HIF-2α translation inhibitor in 0.05% DMSO for 3 h in hypoxia. Control cells were treated with 0.05% DMSO. After the hypoxic exposure, the media was replaced with fresh oxygenated serum-free media containing 5 µg/ml JC-1 for 30 min in atmospheric oxygen. The media were removed, and fresh serum-free media were added to the cells. Control cells incubated with DMSO only were treated in a similar manner. We used serial confocal imaging to monitor mitochondrial membrane depolarization in HLE-B3 cells after treatment with the HIF-1α/HIF-2α double translation inhibitor. Sequential images of a random field of cells were taken every 150 s throughout the 60 min duration (Bar=20 µm). Confocal images of the HIF-1α/HIF-2α double translation inhibitor-treated cells indicated that there was a marked increase in green fluorescence intensity (indicative of depolarization) at both 30 and 60 min (**B**) compared with the control cells (**A**). **C**: HLE-B3 cells treated with the HIF-1α/HIF-2α double translation inhibitor exhibited a significantly increased green/red ratio compared with control, untreated cells.

HLE-B3 cells were treated with 50 μM of the HIF-1α/HIF-2α translation inhibitor, FM19G11, for 3 h in hypoxia. After the hypoxic exposure, the media were replaced with fresh, oxygenated serum-free media containing 5 µg/ml JC-1 for 30 min in atmospheric oxygen. The media were removed, and fresh media without inhibitor were added to the cells. The control cells were incubated in serum-free medium with DMSO. A significant increase in the green-to-red fluorescence ratios, indicating profound mitochondrial depolarization, was observed with the cells treated with the HIF-1α/HIF-2α translation inhibitor compared to control cells (Figure 5).

### Effect of ERK-1/2 and Akt phosphorylation inhibition on HIF-1α, HIF-2α, and downstream VEGF expression in hypoxia

**Figure 6:**
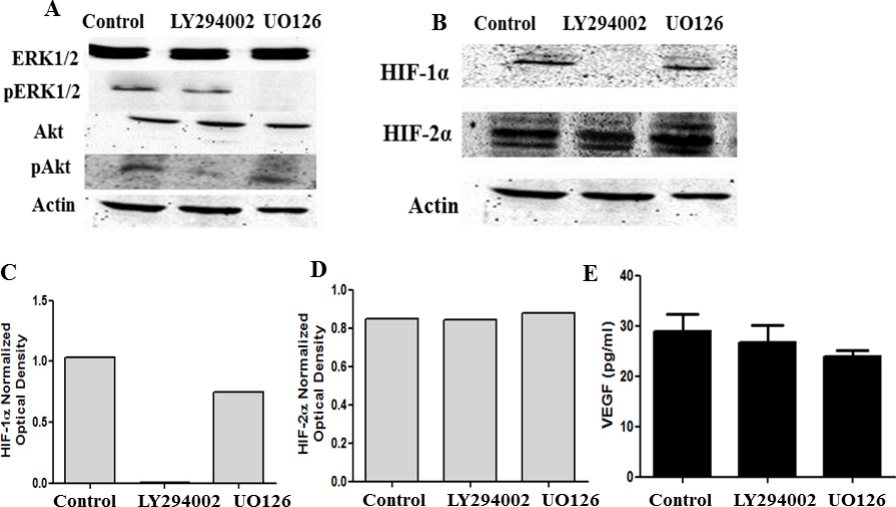
Inhibition of pERK or pAkt does not affect VEGF expression. Western blot analysis of ERK1/2 and Akt phosphorylation inhibition on HIF-1α, HIF-2α, or downstream VEGF expression in hypoxia. HLE-B3 cells were incubated with 25 µM of LY294002 or 10 µM UO126 in 0.05% DMSO for 8 h under hypoxia. Control cells were incubated in serum-free media with 0.05% DMSO. Cell lysates were collected for western blot analysis, and cell-free supernatants were collected for ELISA. LY294002 blocked Akt phosphorylation without interrupting pERK, while UO126 prevented ERK phosphorylation without interfering with pAkt (**A**). Inhibition of Akt phosphorylation suppressed HIF-1α expression, but HIF-2α levels remained unchanged. Inhibition of ERK1/2 phosphorylation did not repress HIF-1α and HIF-2α expression (**B**). **C** and **D** represent the corresponding densitometry analysis. Neither phosphorylation inhibitor diminished VEGF synthesis relative to control cells (**E**). One-way ANOVA analysis was performed to compare the VEGF levels between the control and the LY294002- or UO126-treated samples, and the p value was >0.05.

Previous studies by Yang et al. [28] with human retinal pigment epithelium showed that the PI3-K/Akt pathway, but not the MEK/ERK pathway, is required for the expression of HIF-1α in hypoxia. Cells were treated with 25 µM of the PI3-kinase inhibitor LY294002 and 10 µM of the ERK1/2 inhibitor UO126 in 0.05% DMSO for 8 h in hypoxia. Control cells were incubated in serum-free media with 0.05% DMSO. Inhibiting phosphorylated extracellular signal regulated kinase (pERK) and phosphorylated protein kinase B (pAkt) by the inhibitors was confirmed with western blot analysis (Figure 6A). The cell lysates were analyzed for HIF-1α and HIF-2α expression with western blot analysis. Inhibiting PI3-K in the HLE-B3 cells suppressed HIF-1α expression without altering the HIF-2α levels. Inhibiting ERK1/2 phosphorylation with UO126 did not alter the relative expression of HIF-1α and HIF-2α for cells maintained in hypoxia (Figure 6B). Activation of the mitogen-activated protein kinase/ERK and Akt pathways is known to promote cell survival [29]. Whether these pathways are involved in regulating VEGF synthesis in hypoxia for HLE-B3 cells is not known. To address this question, HLE-B3 cells were treated as described above. Cell-free supernatants were collected in triplicate and analyzed for VEGF with ELISA. Inhibiting ERK1/2 phosphorylation and Akt phosphorylation did not dampen VEGF synthesis in hypoxia in HLE-B3 cells (Figure 6E).

### Cytoprotection in HLE-B3 cells in hypoxia is not affected by inhibition of Akt and ERK-1/2

**Figure 7:**
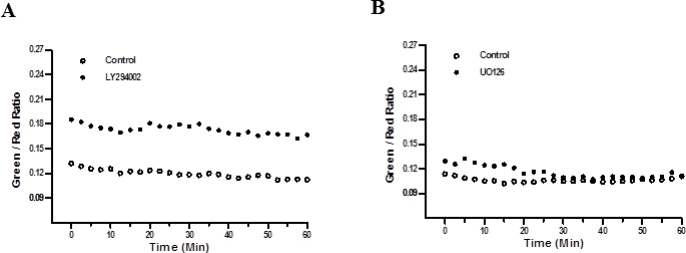
Inhibition of pERK or pAkt does not affect mitochondrial membrane potential. JC-1 analysis of HLE-B3 cells treated with LY294002 or UO126. Cells were initially seeded onto 35 mm dishes and allowed to grow to semiconfluence. The cells were then pretreated with 25 µM LY294002 or 10 µM UO126 in 0.05% DMSO for 3 h in hypoxia to determine the state of mitochondrial membrane potential. Control cells were treated with 0.05% DMSO in serum-free media. After hypoxic exposure, the media were replaced with fresh, oxygenated serum-free media containing 5 µg/ml JC-1 for 30 min in atmospheric oxygen. The media were removed, and fresh serum-free media without inhibitor were added to the cells. Serial confocal imaging indicated no significant difference in the green/red ratio between the cells treated with LY294002 (**A**) or UO126 (**B**) and control cells (Student *t* test, p>0.05).

HLE-B3 cells were treated with 25 µM of LY294002 or 10 µM of UO126 for 3 h in hypoxia. Control cells were treated with 0.05% DMSO. Following the inhibitor treatment, the media were replaced with fresh, oxygenated serum-free media containing 5 µg/ml JC-1 for 30 min in atmospheric oxygen. The media were removed, and fresh media were added to the cells. Inhibition of ERK1/2 and Akt phosphorylation did not initiate mitochondrial depolarization (Figure 7A,B).

### VEGF maintains Bcl-2 expression and promotes cell survival in hypoxia

Previous studies by Beierle et al. [30] have shown that an increase in VEGF levels in neuroblastoma cells increases the expression of prosurvival protein Bcl-2 and protects the cells from apoptotic stimuli. In our study, a significant decrease in VEGF levels coupled with a profound increase in mitochondrial depolarization was observed when the cells were treated with the HIF-1α/HIF-2α translation inhibitor (Figure 4L and Figure 5C). To further evaluate the possible role of VEGF in lenticular cell survival, we examined the association between VEGF levels and the expression of the proapoptotic protein BAX and the prosurvival protein Bcl-2. Lysates were collected from cells treated with various inhibitors (topotecan, HIF-2α (CAS882268–69–1), HIF-1α /HIF-2α (FM19G11), LY294002, and UO126) as described above and analyzed with western blot analysis.

**Figure 8:**
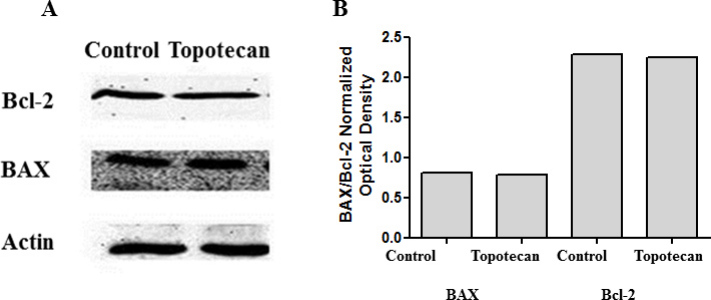
Inhibition of HIF-1α does not influence BAX or Bcl-2 levels. Cell lysates collected from cells treated with 500 nM topotecan were analyzed with western blot analysis for BAX and Bcl-2 levels. There was no change in the protein levels of BAX and Bcl-2 (A). (B) represents the corresponding densitometry analysis.

**Figure 9:**
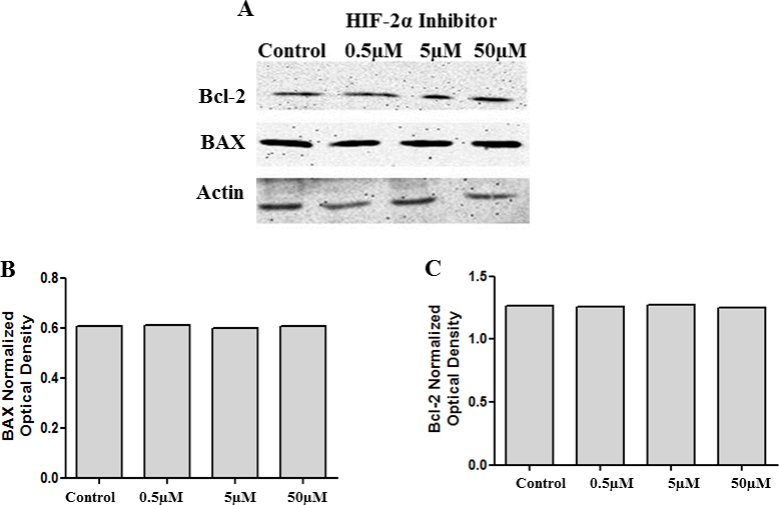
Inhibition of HIF-2α does not influence BAX or Bcl-2 levels. Cell lysates collected from cells treated with 0.5 µM, 5 µM, and 50 µM of HIF-2α translation inhibitor were analyzed with western blot analysis for BAX and Bcl-2 levels. There was no change in the protein levels of BAX and Bcl-2 (**A**). **B** and **C** represent the corresponding densitometry analysis.

**Figure 10:**
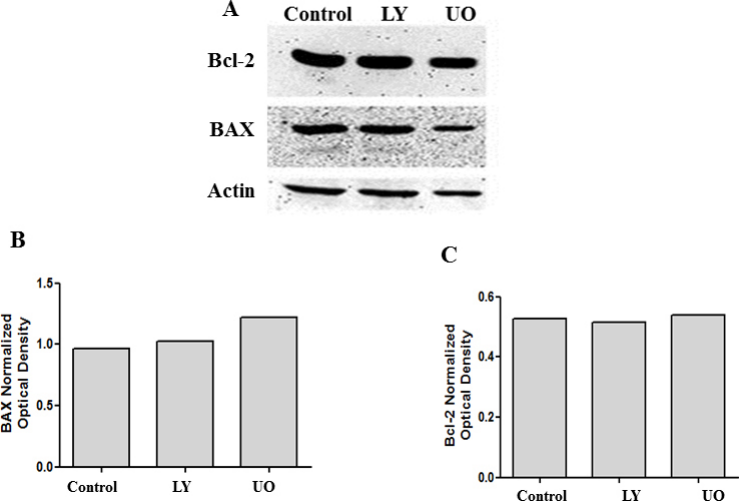
Inhibition of pERK or pAkt does not influence BAX or Bcl-2 levels. Cell lysates collected from cells treated with 25 µM of LY294002 or 10 µM of UO126 were analyzed with western blot analysis for BAX and Bcl-2 levels. There was no change in the protein levels of BAX and Bcl-2 (**A**). **B** and **C** represent the corresponding densitometry analysis.

**Figure 11:**
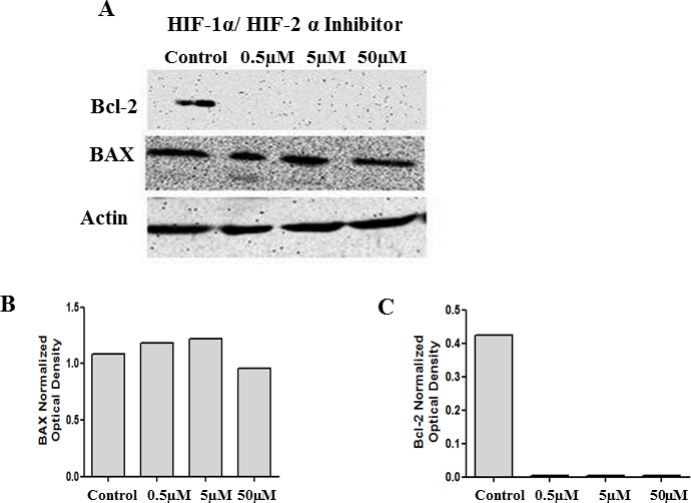
Inhibition of both HIF1α and HIF-2α diminishes Bcl-2 levels without affecting BAX levels. Cell lysates collected from cells treated with 0.5 µM, 5 µM, and 50 µM of HIF-1α/HIF-2α translation inhibitors were analyzed with western blot analysis for BAX and Bcl-2 levels. There was no change in the protein levels of BAX, and a significant decrease in the levels of Bcl-2 coupled with the loss of VEGF (Figure 4L) was observed with the double translation inhibitor (**A**). **B** and **C** represent the corresponding densitometry analysis.

The inhibition of HIF-1α by topotecan and the inhibition of HIF-2α by the HIF-2α translation inhibitor did not alter the levels of VEGF (Figure 2D and Figure 4H) or the apoptotic proteins BAX and Bcl-2 (Figure 8 and Figure 9). Likewise, inhibiting phosphorylation of ERK1/2 and Akt did not alter the levels of VEGF (Figure 6E) or the apoptotic proteins BAX and Bcl-2 (Figure 10). However, the simultaneous inhibition of HIF-1α and HIF-2α that resulted in a significant decrease in the VEGF levels (Figure 4L) diminished the levels of the prosurvival protein Bcl-2, without altering the levels of the proapoptotic protein BAX (Figure 11), and under these conditions profound mitochondrial depolarization was observed (Figure 5C).

### VEGFR tyrosine kinase

**Figure 12:**
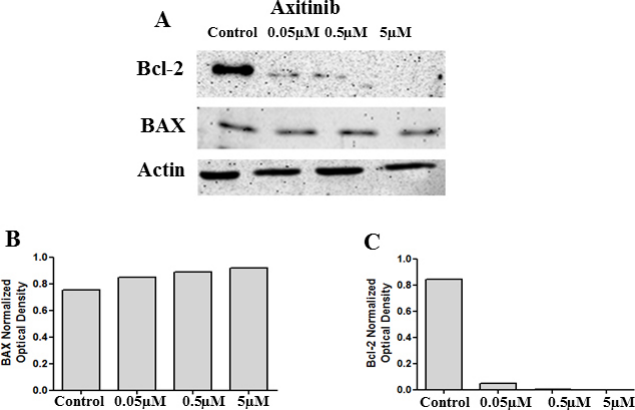
Axitinib blocks binding of VEGF to its receptor, VEGFR2 and prompts a loss of Bcl-2 levels. Cell lysates collected from cells treated with 0.05 µM, 0.5 µM, and 5 µM of axitinib (AG013736) were analyzed with western blot analysis for the BAX and Bcl-2 levels. There was no change in the protein levels of BAX, and a significant decrease in the levels of Bcl-2 was observed with the VEGF receptor inhibitor (**A**). **B** and **C** represent the corresponding densitometry analysis.

Axitinib is a selective receptor tyrosine kinase (RTK) inhibitor that inhibits VEGFR1, VEGFR2, and VEGFR3 [17]. To test the effect of VEGF receptor inhibition on Bcl-2 levels, HLE-B3 cells were incubated with three different concentrations (0.05 µM, 0.5 µM, and 5 µM) of axitinib for 3 h of hypoxia. Control cells were treated with 0.05% DMSO. Cell lysates were collected for western blot analysis of BAX and Bcl-2. Blocking the VEGF RTK activity significantly diminished the levels of the prosurvival protein Bcl-2 (Figure 12), confirming the role of VEGF as a prosurvival factor in the hypoxic adult lens epithelium.

## Discussion

Cells respond to hypoxia by activating several signal transduction mechanisms that promote cell proliferation, cell survival, angiogenesis, and erythropoiesis. Among the activation of these transcription factors are the HIFs. The hypoxia inducible factors, in turn, activate the transcription of various hypoxia responsive genes, including VEGF and EPO. Radreau et al. [31] have shown an increase in the mRNA levels of EPO in FHL-124 lens epithelial cells as induced by HIF-1α. However, whether this increase in mRNA synthesis translates into increased protein levels of EPO was not addressed in their study. Studies in our laboratory agree with those from Radreau et al. [31] in that a tenfold increase in mRNA levels of EPO was noted in hypoxia-treated cells relative to cells maintained in atmospheric oxygen. Germane to our current study, this increase in EPO mRNA did not translate into an increase in protein synthesis. Indeed, no erythropoietin protein expression was detected in cells maintained in either hypoxia or atmospheric oxygen as determined with ELISA (data not shown). Based on these results, we conclude that EPO does not play a role in the lenticular cytoprotection of adult, cultured human lens epithelial cells.

To date, published studies support a role for HIF-1α as a transcription factor that controls VEGF expression in hypoxia, but there are inconsistencies in the lens literature. HIF-1 is recognized as an age-dependent regulator of lens cell proliferation in the hypoxic lens and is known to degrade under conditions in or above atmospheric oxygen [6]. However, studies have been demonstrated that a baseline level of VEGF is continuously synthesized and accumulates in atmospheric oxygen, under conditions where HIF-1α is degraded [31]. Additionally, Garcia et al. [7] demonstrated that VEGF continues to be synthesized in the hypoxic lens in the absence of HIF-1. In other words, there is continuous expression of VEGF, in lens or cultured lens epithelial cells, whether it is in hypoxia or atmospheric oxygen, in the absence of HIF-1α, suggesting that additional regulatory proteins must also be at play.

In this study, we have established the finding that the survival of human lens epithelial cells in hypoxia depends on the uninterrupted and sustained synthesis of VEGF levels controlled by the expression of either of the hypoxia inducible factors, HIF-1α or HIF-2α. The novelty of the finding is that a decrease in the manifestation of HIF-1α may be compensated for by HIF-2α accumulation and vice versa. That is, the naturally hypoxic lens epithelium regulates VEGF levels via the coordinated adjustment of two hypoxia inducible factors, HIF-1α and HIF-2α. By using a combination of pharmacological and metabolic inhibitors, we established the nature of the compensatory relationship between the expression of the hypoxia inducible factors and their association with VEGF expression. With topotecan, a specific topoisomerase I inhibitor, which inhibits HIF-1α protein accumulation and therefore its transcriptional activity or a specific HIF-1α translation inhibitor (KC7F2), or the Akt phosphorylation inhibitor, LY294002, HIF-1α was shown to decrease without affecting the levels of VEGF (Figure 2D, Figure 4D, and Figure 6E). Under this condition, there was no depolarization (Figure 3 and Figure 7A), and HIF-2α was continuously expressed (Figure 2A, Figure 4A, and Figure 6B). With a specific HIF-2α translation inhibitor (CAS882268–69–1), HIF-2α decreased without affecting the levels of VEGF (Figure 4H), and HIF-1α was continuously expressed. The data suggest a compensatory role for HIF-1α and HIF-2α in maintaining continuous VEGF expression. However, the use of the HIF-1α/HIF-2α double translation inhibitor (FM19G11) diminished the expression of both hypoxia inducible factors (Figure 4I), resulting in a significant reduction in the VEGF levels (Figure 4L) and induced profound mitochondrial depolarization (Figure 5). These data support our contention that sustained VEGF expression plays a vital cytoprotective role in preventing hypoxic lens epithelial cells from entering the cell death pathway and, moreover, suggests a possible mechanism for how the naturally hypoxic lens epithelium thrives in low oxygen.

VEGF is expressed in nearly every adult tissue, and although much is known regarding the role of VEGF in angiogenesis and the factor’s function as a cell survival protein in other ocular tissues, little is known about the role of VEGF as a survival factor in the human lens epithelium. VEGF is generally produced as three isoforms in mice, VEGF 120, VEGF 164, and VEGF 188. Using embryos from VEGF lacZ mice, Saint-Geniez et al. [32] demonstrated that VEGF 164 is the major isoform in embryonic lens. Transgenic mice expressing only the VEGF 120 isoform showed major defects in eye development including failed lens differentiation. An essential role for retinal pigment epithelium (RPE)-derived VEGF in maintaining the choriocapillaris was shown by Saint-Geniez et al. [33] using mice that produced only VEGF 188 (an insoluble form of VEGF). The study demonstrated that VEGF 188/188 mice exhibited normal early development of the choriocapillaris. However, by 7 months of age (by then, a fully developed Bruch’s membrane is in place), progressive degeneration of the choriocapillaris was reported. That is, as diffusion of VEGF 188 across Bruch’s membrane became more limited, clear atrophy of the choriocapillaris was noted. Another study by Ford et al. [34], using bevacizumab to neutralize VEGF demonstrated that RPE became progressively more vacuolated and separated from photoreceptor outer segments, and choriocapillaris fenestrations were decreased. These results indicate that VEGF plays a critical role in the survival and maintenance of RPE integrity. In this study, we have shown a positive association between sustained VEGF levels and lenticular cytoprotection, suggesting the role of VEGF as a prosurvival factor in the naturally hypoxic adult lens.

Saint-Geniez et al. [32] observed VEGFR2 in the lens epithelium and differentiating lens fibers of the marginal zone of the embryonic mouse. Shui et al. [5] also demonstrated the presence of VEGFR2 (and VEGF) in adult mouse and human eyes in lens epithelium and fiber cells. It is likely that within the natural lens, the hypoxic response described in the current study may be relegated, to greater or lesser extent, to the immediate subjacent lens fiber cells in juxtaposition to the anterior epithelium and the differentiating marginal fiber cells, as well as the anterior epithelium. Our data with anterior lens epithelial cell cultures do not permit the distinction or weight of the contribution of one zone (i.e., epithelium versus fiber cells) over the other regarding the hypoxic response.

Axitinib is a potent inhibitor of VEGFR1, VEGFR2, and VEGFR3 [17,35-37]. Our data with axitinib reconfirm our observation with the HIF-1α/HIF-2α double translation inhibitor (Figure 11) in that the blocking of the autophosphorylation of VEGFR2 prevents the interaction of VEGF with its receptor, thus functioning as a “loss” of VEGF from the cell system resulting in a loss of expression of Bcl-2. But more than that, our observation demonstrates the biologic significance of VEGF signaling for lens epithelial cell survival and the role played by the VEGF-VEGFR2 complex in the signaling pathway in which VEGF regulates endogenous Bcl-2 levels. It is tempting to suggest a potential mechanism in which the synthesized and secreted VEGF interacts with cell surface VEGFR2 in an autocrine fashion, which then initiates the downstream cell survival pathway. It must be stressed, however, that axitinib is likely to be a cell membrane permeable compound and that our data do not rule out the possibility that the VEGF-VEGFR2 complex first undergoes intracellular compartmentalization and that axitinib inhibits VEGFR2 signaling via an endogenous mechanism. This, of course, would likely be in conjunction with inhibiting VEGFR autophosphorylation as mediated by the secreted, extracellular VEGF in an autocrine fashion. Our data support the fact that these survival signals are triggered by the stress situation generated by hypoxia. Moreover, we must also point out that our data cannot rule out the possibility that the lens is also influenced by paracrine activation that may also influence the VEGF-VEGFR2 complex. As VEGF may also be secreted by the ciliary process [38], we cannot exclude the possibility that paracrine VEGF signaling plays some role in hypoxic lens survival function.

Cellular apoptosis is regulated, in part, by maintaining a balance between the levels of the prosurvival protein Bcl-2 and the proapoptotic protein BAX. BAX is a proapoptotic member of the Bcl-2 family. Activation of BAX causes its translocation to the mitochondrial outer membrane and induces mitochondrial depolarization, but before BAX can initiate this process, the protein must first translocate from the cytoplasm to the mitochondria. Using lenses isolated from E10 chick embryo fractions, Weber et al. [39] demonstrated that an increase in Bcl-2 expression during lens differentiation binds to BAX and “helps in the controlled release of cytochrome C from mitochondria without tipping the balance toward apoptosis.” Indeed, Nomura et al. [40] showed that Bcl-2 inhibits the translocation of BAX from the cytoplasm to mitochondria and thus prevents apoptosis from being initiated. Others have shown that VEGF protects endothelial cells from apoptotic stimuli by increasing Bcl-2 expression [41]. In this study, inhibition of either HIF-1α or HIF-2α or the phosphorylation of ERK and Akt using specific pharmacological inhibitors did not have an effect on either the VEGF levels (Figure 2D, Figure 4H, and Figure 6E) or Bcl-2 protein expression (Figure 8, Figure 9, and Figure 10). Using a specific HIF-1α/HIF-2α double translation inhibitor, we have demonstrated that a decrease in VEGF levels resulted in markedly diminished Bcl-2 levels (Figure 11). The loss of Bcl-2 associated with a significant increase in mitochondrial depolarization (Figure 5C). The use of axitinib, which blocked the VEGF–VEGFR2 receptor complex, reconfirmed our observations with the HIF-1α/HIF-2α double translation inhibitor, in that consistent expression of VEGF is required for regulating endogenous levels of Bcl-2, thus avoiding entry into the mitochondrial cell death pathway (Figure 12).

We have yet to delve into the precise mechanism by which VEGF–VEGFR2 regulates downstream synthesis of Bcl-2 in the hypoxic lens epithelium. In this study, we have demonstrated that inhibiting PI3-kinase and ERK1/2 phosphorylation did not influence Bcl-2 levels (Figure 10) when the lens cells are maintained in hypoxia.

Our data suggest a potential mechanism by which the naturally hypoxic lens epithelium survives in low oxygen and resists cell death. That mechanism entails sustained VEGF synthesis in hypoxia, which is tightly regulated by the hypoxia inducible factors, HIF-1α and HIF-2α. Sustained VEGF expression maintains a consistent level of Bcl-2. Any reduction in the levels of intracellular VEGF, in association with the loss of Bcl-2, is likely to permit the translocation of BAX from the cytosol to the mitochondria and induce mitochondrial depolarization.

Our studies to date have been performed solely with virally transformed human lens epithelial cells. We cannot, at this time, be certain that the state of viral transformation may influence the expression of the hypoxia inducible factors-1α and -2α. Although this remains to be determined, we believe that the nature of the VEGF-VEGFR2 complex and the role of VEGF in lens epithelial cell survival in hypoxia will be faithfully reproduced with “normal” lens epithelial cell cultures. To this end, we have committed all future laboratory efforts to demonstrate this pathway using bovine lens epithelial cell explants.
